# Incidence and prevalence of sporadic and hereditary MTC in Denmark 1960–2014: a nationwide study

**DOI:** 10.1530/EC-18-0157

**Published:** 2018-05-14

**Authors:** Jes Sloth Mathiesen, Jens Peter Kroustrup, Peter Vestergaard, Kirstine Stochholm, Per Løgstrup Poulsen, Åse Krogh Rasmussen, Ulla Feldt-Rasmussen, Sten Schytte, Stefano Christian Londero, Henrik Baymler Pedersen, Christoffer Holst Hahn, Bjarki Ditlev Djurhuus, Jens Bentzen, Sören Möller, Mette Gaustadnes, Maria Rossing, Finn Cilius Nielsen, Kim Brixen, Anja Lisbeth Frederiksen, Christian Godballe, the Danish Thyroid Cancer Group (DATHYRCA)

**Affiliations:** 1 Department of ORL Head & Neck SurgeryOdense University Hospital, Odense, Denmark; 2 Department of Clinical ResearchUniversity of Southern Denmark, Odense, Denmark; 3 Department of Clinical Medicine and EndocrinologyAalborg University Hospital, Aalborg, Denmark; 4 Department of Internal Medicine and EndocrinologyAarhus University Hospital, Aarhus, Denmark; 5 Center for Rare DiseasesAarhus University Hospital, Aarhus N, Denmark; 6 Department of Medical EndocrinologyCopenhagen University Hospital, Copenhagen, Denmark; 7 Department of ORL Head & Neck SurgeryAarhus University Hospital, Aarhus, Denmark; 8 Department of ORL Head & Neck SurgeryAalborg University Hospital, Aalborg, Denmark; 9 Department of ORL Head & Neck SurgeryCopenhagen University Hospital, Copenhagen, Denmark; 10 Department of ORL Head & Neck SurgeryZealand University Hospital, Køge, Denmark; 11 Department of OncologyHerlev Hospital, Herlev, Denmark; 12 Odense Patient data Explorative Network (OPEN)Odense University Hospital, Odense, Denmark; 13 Department of Molecular MedicineAarhus University Hospital, Aarhus, Denmark; 14 Center for Genomic MedicineCopenhagen University Hospital, Copenhagen, Denmark; 15 Department of Clinical GeneticsOdense University Hospital, Odense, Denmark

**Keywords:** sporadic medullary thyroid carcinoma, hereditary medullary thyroid carcinoma, incidence, prevalence, Denmark

## Abstract

Recent studies have shown a significant increase in the temporal trend of medullary thyroid carcinoma (MTC) incidence. However, it remains unknown to which extent sporadic medullary thyroid carcinoma (SMTC) and hereditary MTC (HMTC) affect the MTC incidence over time. We conducted a nationwide retrospective study using previously described *RET* and MTC cohorts combined with review of medical records, pedigree comparison and relevant nationwide registries. The study included 474 MTC patients diagnosed in Denmark between 1960 and 2014. In the nationwide period from 1997 to 2014, we recorded a mean age-standardized incidence of all MTC, SMTC and HMTC of 0.19, 0.13 and 0.06 per 100,000 per year, respectively. The average annual percentage change in incidence for all MTC, SMTC and HMTC were 1.0 (*P* = 0.542), 2.8 (*P* = 0.125) and −3.1 (*P* = 0.324), respectively. The corresponding figures for point prevalence at January 1, 2015 were 3.8, 2.5 and 1.3 per 100,000, respectively. The average annual percentage change in prevalence from 1998 to 2015 for all MTC, SMTC and HMTC was 2.8 (*P* < 0.001), 3.8 (*P* < 0.001) and 1.5 (*P* = 0.010), respectively. We found no significant change in the incidence of all MTC, SMTC and HMTC possibly due to our small sample size. However, due to an increasing trend in the incidence of all MTC and opposing trends of SMTC (increasing) and HMTC (decreasing) incidence, it seems plausible that an increase for all MTC seen by others may be driven by the SMTC group rather than the HMTC group.

## Introduction

Medullary thyroid carcinoma (MTC) is a neuroendocrine tumor arising from the calcitonin secreting parafollicular C cells of the thyroid gland. MTC is divided into sporadic MTC (SMTC) and hereditary MTC (HMTC) accounting for approximately 75% and 25%, respectively. HMTC occurs as part of the autosomal dominant inherited cancer syndromes, multiple endocrine neoplasia (MEN) 2A and MEN2B. MEN2A and MEN2B account for approximately 95% and 5% of all MEN2 patients, respectively. MEN2A associates MTC, pheochromocytoma (PHEO), hyperparathyroidism (HPTH), cutaneous lichen amyloidosis and Hirschsprung’s disease, while MEN2B associates MTC, PHEO, ganglioneuromatosis of the aerodigestive tract, and facial, ophthalmologic and skeletal abnormalities. Both syndromes are caused by germline mutations of the *RE*arranged during *T*ransfection (*RET*) proto-oncogene (1, 2).

Recent studies have shown a significant increase in the temporal trend of MTC incidence (3, 4, 5, 6, 7, 8). However, it remains unknown to which extent SMTC and HMTC affect the MTC incidence over time.

Consequently, we conducted the first nationwide study aiming to assess the significance of SMTC and HMTC in regards to the time trends in MTC incidence. Additionally, we describe prevalence changes over time.

## Patients and methods

### Patients

This retrospective cohort study included 474 unique patients diagnosed with MTC in Denmark between January 1, 1960 and December 31, 2014. Of these, 356 were classified as SMTC and 113 as HMTC. Five were left unclassified.

An MTC cohort, initially comprising 476 patients diagnosed with MTC in Denmark between January 1960 and December 2014, was constructed through three nationwide registries: the Danish Thyroid Cancer (DATHYRCA) Database, the Danish Cancer Registry and the Danish Pathology Register (9, 10, 11). This has been described in detail elsewhere (12). We only included patients with a histological or cytological MTC diagnosis, which was the case for 474 and 2 patients, respectively. Two of the 476 MTC patients were excluded as they were diagnosed in Denmark, while being inhabitants of the Faroe Islands. This resulted in 474 MTC patients eligible for the study.

**Figure 1:**
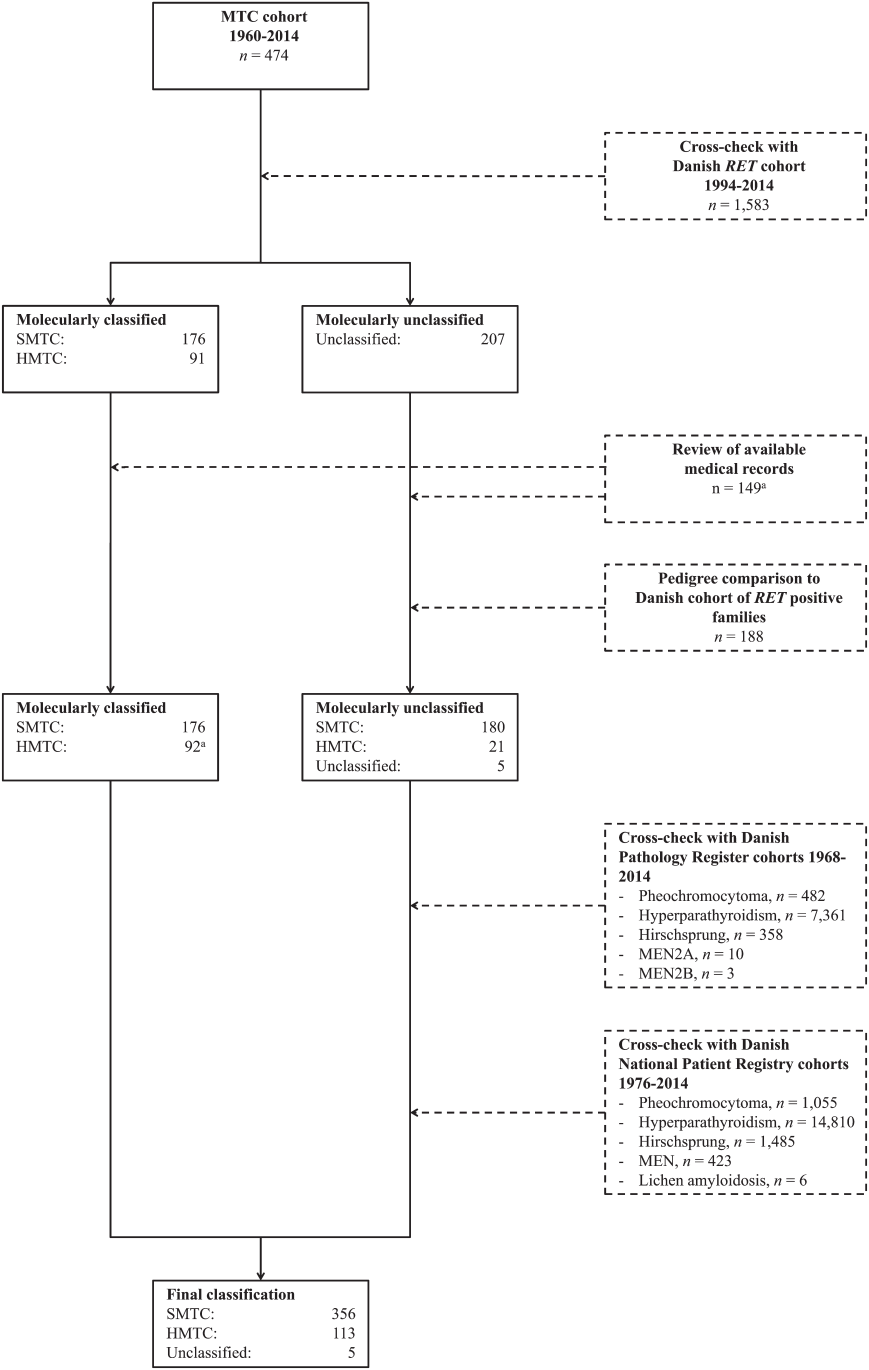
Flow chart showing 474 patients with medullary thyroid carcinoma classified into the sporadic or hereditary type. Dotted boxes indicate methods used. MTC, medullary thyroid carcinoma; SMTC, sporadic MTC; HMTC, hereditary MTC; *RET*, rearranged during transfection; MEN, multiple endocrine neoplasia. ^a^One patient *RET* tested subsequent to the end date of the *RET* cohort in December 31, 2014.

In order to molecularly classify the MTC patients as having either SMTC or HMTC, the MTC cohort was cross-checked with the nationwide Danish *RET* cohort containing all patients (*n* = 1583) *RET* tested in Denmark from September 1994 to December 2014. The *RET* cohort has been described in detail previously (13). Patients were classified as HMTC if tested positive for a *RET* sequence change classified as pathogenic in the ARUP MEN2 database on April 1, 2018 (14). If tested negative, patients were classified as SMTC. Cross-check between the MTC and *RET* cohort revealed that 272 of the 474 MTC patients had been *RET* tested, while 202 patients had not been tested. Five of the 272 patients were tested by a method for detecting the C611Y mutation only (15). Consequently, 207 MTC patients were not adequately molecularly classified. Among the 267 MTC patients eligible for molecular classification, 91 and 176 were classified as HMTC and SMTC, respectively (Fig. 1).

To classify the 207 molecularly unclassified MTC patients, several sources were used.

First, a review of available medical records (*n* = 149) was undertaken. This revealed that one patient had been tested positive for the C634R mutation subsequent to the end date of the *RET* cohort December 31, 2014, and thus reduced the number of molecularly unclassified patients to 206. The remaining patients were classified as HMTC, if there was presence of: 1) a family history of MEN2 (MTC, PHEO, HPTH, Hirschsprung’s disease, cutaneous lichen amyloidosis, mucosal neuromas/ganglioneuromatosis) or 2) a MEN2 feature (histologically verified PHEO, Hirschsprung’s disease, clinically diagnosed cutaneous lichen amyloidosis or mucosal neuromas/ganglioneuromatosis, or histologically and biochemically diagnosed HPTH) other than their MTC. Patients were classified as apparently SMTC, if there was no presence of MEN2 family history and no MEN2 features other than their MTC.

Secondly, relatedness to a nationwide cohort of *RET* positive MEN2 families was assessed through pedigree comparison. This was performed to improve classification, as *RET* germline mutations have been reported in 1.5–14.9% of patients, who have been classified as apparently SMTC in absence of MEN2 family history or other MEN2 features (16, 17, 18, 19, 20, 21, 22). Feasibility of this exercise was based on data indicating that *de novo* mutations occur in only 5.6–9% of MEN2A patients (23), leading to the assumption of a high likelihood of relatedness to our nationwide cohort of *RET*-positive MEN2A families, if a patient had HMTC. From the nationwide Danish *RET* cohort, 36 *RET* positive families were identified (13). Five families (one MEN2A and four MEN2B (three of which have been described elsewhere (24, 25, 26, 27)) were excluded, as mutations of the index patient had been molecularly proven as *de novo*. One family was excluded, as the pathogenicity of the *RET* I852M variant recently has been questioned and re-classified in the ARUP MEN2 database (14, 28). Three families (C634Y, C634Y/Y791F and L790F) were excluded, due to origin outside Denmark. Meanwhile, we included the family of the aforementioned C634R patient. This yielded 28 *RET*-positive families (Table 1). Several of these have been studied earlier (29, 30, 31, 32, 33). A pedigree for each *RET*-positive family was created with a minimum of four generations by use of the Civil Registration System (www.cpr.dk) and the Danish National Archives (www.sa.dk/en/). Similarly, a pedigree for each of the 206 molecularly unclassified MTC patients was created. Four-generation pedigrees were created for 154 patients. Three-, two- and one-generation pedigrees were created for 16, 18 and 18 patients, respectively. Once an individual was born abroad or as an illegitimate child, their ancestors often could not be traced. This was the case in all but three pedigrees where four generations could not be reached. All pedigrees with >1 generation (*n* = 188) were compared to the pedigrees of the 28 *RET*-positive families. If relatedness between an MTC patient and a *RET*-positive family could be proven, the MTC patient was considered to carry the family *RET* mutation, and thus classified as HMTC. If no relatedness, patients were classified as SMTC.

**Table 1:** Families with detected *RET* germline mutations^a^ and Danish origin included in this study.

Family no.	Exon	Nucleotide change	Sequence change	*RET*+^b^/*RET*−	Reference
1	10	c.1833C>G	C611W	6/11	
2	10	c.1832G>A	C611Y	2/0	(29)
3	10	c.1832G>A	C611Y	1/0	
4	10	c.1832G>A	C611Y	8/3	
5	10	c.1832G>A	C611Y	15/13	
6	10	c.1832G>A	C611Y	2/0	(32)
7	10	c.1832G>A	C611Y	9/7	(32)
8	10	c.1832G>A	C611Y	2/6	
9	10	c.1832G>A	C611Y	26/27	
10	10	c.1832G>A	C611Y	30/30	(30)
11	10	c.1832G>A	C611Y	1/3	
12	10	c.1832G>A	C611Y	5/18	(31)
13	10	c.1832G>A	C611Y	7/8	
14	10	c.1853G>T	C618F	1/1	
15	10	c.1853G>T	C618F	2/1	(32)
16	10	c.1853G>A	C618Y	5/9	
17	10	c.1853G>A	C618Y	3/3	(32)
18	10	c.1858T>C	C620R	6/5	(33)
19	10	c.1858T>C	C620R	3/3	(32)
21	11	c.1891G>T	D631Y	1/0	
22	11	c.1900T>C	C634R	2/0	
23	11	c.1900T>C	C634R	1/2	(32)
24	11	c.1900T>C	C634R	3/11	(30)
25	11	c.1900T>C	C634R	1/5	(32)
29	14	c.2410G>A	V804M	2/1	
34	16	c.2753T>C	M918T	1/2	
36	16	c.2753T>C	M918T	1/1	
37	11	c.1900T>C	C634R	1/0	

Modified from Table 2 of Mathiesen *et al.* (13).

^a^Sequence changes classified as pathogenic in the ARUP MEN2 database April 1, 2018 (14); ^b^*RET*+ includes index cases.

*RET*, **rearranged during transfection.

When using both medical records and pedigree comparison to classify the 206 molecularly unclassified patients, 21 and 180 fulfilled the criteria for HMTC and SMTC, respectively (Table 2). Five patients could not be classified, as medical records were unavailable and pedigrees with >1 generation could not be created (Fig. 1).

**Table 2:** Methods and criteria used for classification of 474 patients with medullary thyroid carcinoma in Denmark 1960–2014.

Category	Methods	Criteria	1960–1996 (*n* (%))	1997–2014 (*n* (%))	1960–2014 (*n* (%))
SMTC	*RET* testing	No *RET* mutation^a^ detected	47 (19)	129 (58)	176 (37)
	Pedigree comparison and medical record review	No relatedness to *RET* positive family^b^ and no MEN2 feature^c^ other than MTC and no presence of MEN2 family history^d^	79 (32)	37 (17)	116 (24)
	Pedigree comparison only	No relatedness to *RET* positive family	49 (20)	2 (1)	51 (11)
	Medical record review only	No MEN2 feature other than MTC and no presence of MEN2 family history	10 (4)	3 (1)	13 (3)
Total			185 (74)	171 (76)	356 (75)
HMTC	*RET* testing	*RET* mutation detected	39 (16)	53 (24)	92 (19)
	Pedigree comparison and medical record review	Relatedness to *RET* positive family and/or MEN2 feature other than MTC and/or presence of MEN2 family history	15^e^ (6)	0 (0)	15^e^ (3)
	Pedigree comparison only	Relatedness to *RET* positive family	2^e^ (1)	0 (0)	2^e^ (0)
	Medical record review only	MEN2 feature other than MTC and no presence of MEN2 family history	4^f^ (2)	0 (0)	4^f^ (1)
Total			60 (24)	53 (24)	113 (24)
Unclassified			5 (0)	0 (0)	5 (1)
All			250 (100)	224 (100)	474 (100)

Due to rounding up, not all sums of percentages fit.

^a^Sequence changes classified as pathogenic in the ARUP MEN2 database April 1, 2018 (14); ^b^*RET* positive family defined as a family from Table 1; ^c^MEN2 feature defined as histologically verified pheochromocytoma, Hirschsprung’s disease, clinically diagnosed cutaneous lichen amyloidosis or mucosal neuromas/ganglioneuromatosis, or histologically and biochemically diagnosed hyperparathyroidism; ^d^MEN2 family history defined as history of MTC, pheochromocytoma, hyperparathyroidism, Hirschsprung’s disease, cutaneous lichen amyloidosis or mucosal neuromas/ganglioneuromatosis; ^e^Due to relatedness to *RET* positive families, all 17 patients were considered *RET* mutation carriers: one C611W, twelve C611Y, one 618Y, one D631Y, one 634R and one V804M; ^f^All patients had phenotypically MEN2B and have been described elsewhere (12).

HMTC, hereditary MTC; MEN2, multiple endocrine neoplasia 2; MTC, medullary thyroid carcinoma; *RET*, rearranged during transfection; SMTC, sporadic MTC.

Thirdly, the cohort of molecularly unclassified MTC patients was cross-checked with relevant cohorts identified through two nationwide registries: Danish Pathology Register and the Danish National Patient Registry (11, 34). Details can be seen in Supplementary Material (see section on supplementary data given at the end of this article). This was carried out to identify MEN2 features in MTC patients without medical records and to ensure that MEN2 features besides MTC had not been overlooked in the patients with available medical records. With this exercise, we depleted all register-based possibilities for classification, but revealed no HMTC patients, not classified already by medical records and pedigree comparison (Fig. 1).

Therefore, we ended up with 356 SMTC, 113 HMTC and five unclassified. Table 2 shows the methods and criteria used for classification according to time periods.

The investigation was approved by the Danish Health Authority (3-3013-395/3) and the Danish Data Protection Agency (18/17801).

### Methods

The MTC cohort was based on the Danish Cancer Registry, the Danish Pathology Register and the Danish Thyroid Cancer Database, which have prospectively collected data since 1943, 1968 and 1996, respectively. The first year, in which registration was mandatory in all three registries simultaneously, was 1997. This led us to subdivide the inclusion period into an uncertain period (1960–1996) where complete coverage could not be guaranteed, and a nationwide period (1997–2014) where coverage of the entire country was considered complete. In this paper, we primarily focus on the latter period.

### Incidence

Incidence was calculated as the number of all MTC, SMTC and HMTC patients diagnosed per year divided by the number of inhabitants alive in the corresponding year. To ease comparison, incidence standardization was performed according to the World (WHO 2000–2025), the 2000 USA, the European (Scandinavian 1960), the World (Segi 1960) and the 1970 Swedish population. Population data and weights were retrieved from the National Cancer Institute (www.seer.cancer.gov/stdpopulations/) and Statistics Sweden (www.scb.se/en/).

The population at risk (inhabitants alive in Denmark) was roughly constant throughout the years used for incidence calculations. Danish population data were supplied by Statistics Denmark (www.statbank.dk).

### Prevalence

Point prevalence for each year from 1961 to 2015 was calculated as the number of all MTC, HMTC and SMTC patients alive at January 1st divided by the number of inhabitants alive at the same date.

### Statistical analysis

Age at diagnosis was normally distributed in all groups and reported as mean with 95% CI. Direct standardization was used to age-standardize incidences to standard populations. Poisson regression models were applied to estimate time trends in incidence and prevalence by annual percentage change, while the Student’s *t*-test was used for comparison of means. *P* values below 0.05 were considered significant. All analyses were done using Stata 14.2 (StataCorp, USA).

## Results

Demographics and genetic characteristics are described in Table 3. In the nationwide period from 1997 to 2014, the female-to-male ratio for all MTC, SMTC and HMTC were 1.43 (95% CI: 1.05–1.82), 1.63 (95% CI: 1.13–2.13) and 0.96 (95% CI: 0.44–1.48), respectively. In the same period, the mean age at diagnosis for all MTC patients was 52.4 (95% CI: 49.9–54.8) years, while a significant difference was identified between the SMTC and HMTC group (*P* < 0.001).

**Table 3:** Demographic and genetic characteristics of 474 patients with medullary thyroid carcinoma in Denmark, 1960–2014.

Category	1960–1996	1997–2014	1960–2014
SMTC			
Total	185	171	356
Female:male	104:81	106:65	210:146
Mean age at diagnosis, years (95% CI)	57.9 (55.5–60.3)	57.1 (54.7–59.4)	57.5 (55.8–59.2)
Diagnosed by autopsy	13	4	17
HMTC			
Total	60	53	113
Female:male	28:32	26:27	54:59
Mean age at diagnosis, years (95% CI)	44.8 (40.3–49.4)	37.2 (32.4–42.0)	41.2 (37.9–44.6)
Diagnosed by autopsy	1	0	1
*RET* mutation carriers			
C611W	1	3	4
C611Y	40	31	71
C618F	1	1	2
C618Y	2	3	5
C620R	3	4	7
D631Y	1	0	1
C634R	7	1	8
C634R + Y791F	0	1	1
L790F	0	1	1
V804M	1	1	2
A883F	0	1	1
M918T	0	6	6
Unknown	4^a^	0	4^a^
Unclassified			
Total	5	0	5
Female:male	3:2		3:2
Mean age at diagnosis, years (95% CI)	59.0 (47.2–70.8)		59.0 (47.3–70.8)
Diagnosed by autopsy	1		1

Figures indicate number of patients unless otherwise stated.

^a^All patients had phenotypically MEN2B and have been described elsewhere (12).

HMTC, hereditary medullary thyroid carcinoma; *RET*, rearranged during transfection; SMTC, sporadic medullary thyroid carcinoma.

### Incidence

The mean and annual age-standardized (World (WHO 2000–2025)) incidences of all MTC, SMTC and HMTC in the nationwide period are shown in Table 4.

**Table 4:** Mean and annual age-standardized (WHO 2000–2025) incidence of medullary thyroid carcinoma per 100,000 in Denmark, 1997–2014.

Year	All MTC			SMTC			HMTC		
	Both sexes	Female	Male	Both sexes	Female	Male	Both sexes	Female	Male
1997	0.21	0.20	0.22	0.10	0.14	0.06	0.11	0.05	0.16
1998	0.23	0.26	0.20	0.14	0.12	0.16	0.09	0.13	0.05
1999	0.13	0.14	0.12	0.08	0.08	0.08	0.05	0.06	0.03
2000	0.31	0.40	0.23	0.16	0.20	0.11	0.15	0.19	0.12
2001	0.17	0.29	0.06	0.15	0.26	0.06	0.02	0.04	0.00
2002	0.08	0.15	0.02	0.08	0.15	0.02	0.00	0.00	0.00
2003	0.12	0.14	0.11	0.10	0.14	0.06	0.02	0.00	0.05
2004	0.21	0.23	0.19	0.17	0.23	0.13	0.03	0.00	0.07
2005	0.16	0.09	0.22	0.06	0.06	0.06	0.10	0.03	0.16
2006	0.11	0.14	0.08	0.07	0.06	0.08	0.04	0.08	0.00
2007	0.21	0.29	0.13	0.21	0.29	0.13	0.00	0.00	0.00
2008	0.17	0.22	0.13	0.11	0.13	0.09	0.07	0.09	0.05
2009	0.08	0.12	0.05	0.07	0.09	0.05	0.02	0.03	0.00
2010	0.14	0.17	0.10	0.09	0.17	0.00	0.05	0.00	0.10
2011	0.29	0.29	0.28	0.15	0.17	0.13	0.14	0.13	0.15
2012	0.24	0.24	0.24	0.17	0.16	0.17	0.07	0.08	0.07
2013	0.19	0.20	0.18	0.16	0.14	0.18	0.03	0.06	0.00
2014	0.28	0.35	0.22	0.26	0.33	0.19	0.02	0.02	0.03
Mean (95% CI)									
Including autopsy cases	0.19 (0.16–0.21)	0.22 (0.18–0.26)	0.16 (0.12–0.19)	0.13 (0.11–0.15)	0.16 (0.13–0.20)	0.10 (0.08–0.13)	0.06 (0.04–0.07)	0.05 (0.03–0.08)	0.06 (0.04–0.08)
Excluding autopsy cases	0.18 (0.16–0.21)	0.22 (0.18–0.26)	0.15 (0.12–0.19)	0.13 (0.11–0.15)	0.16 (0.13–0.20)	0.09 (0.07–0.12)	0.06 (0.04–0.07)	0.05 (0.03–0.08)	0.06 (0.04–0.08)

HMTC, hereditary MTC; MTC, medullary thyroid carcinoma; SMTC, sporadic MTC.

The age-standardized (World (WHO 2000–2025)) incidence of all MTC increased from 0.21 per 100,000 in 1997 to 0.28 per 100,000 in 2014, corresponding to an average annual percentage change of 1.0 (95% CI: −2.2 to 4.4; *P* = 0.542). In the same period, the average annual percentage change for SMTC and HMTC was 2.8 (95% CI: −0.8 to 6.6; *P* = 0.125) and −3.1 (95% CI: −9.0 to 3.2; *P* = 0.324), respectively (Supplementary Figure 1). Similar non-significant time trends were seen when age-standardizing to the other standard populations.

### Prevalence

The point prevalence at January 1, 2015, for all MTC, SMTC and HMTC was 3.8 (95% CI: 3.3–4.3), 2.5 (95% CI: 2.1–2.9) and 1.3 (95% CI: 1.0–1.6) per 100,000, respectively (215 MTC, 141 SMTC and 74 HMTC patients and 5,659,715 inhabitants).

**Figure 2:**
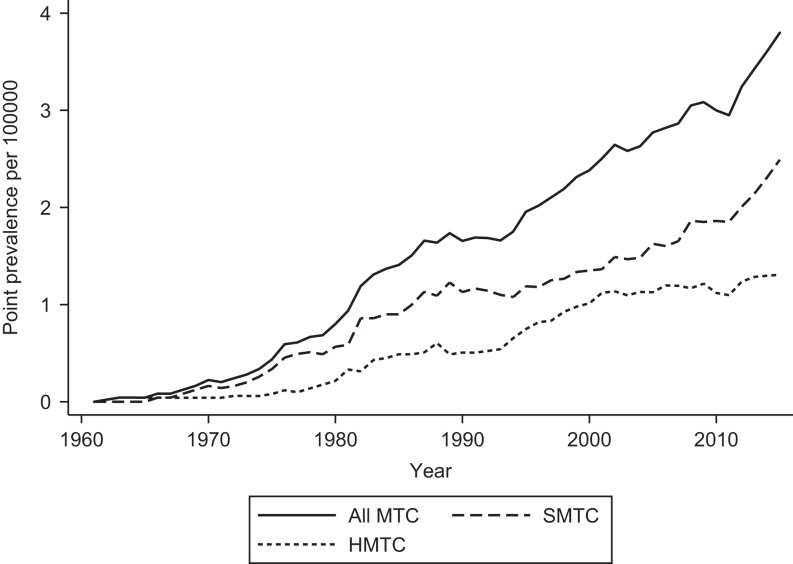
Point prevalence of medullary thyroid carcinoma per 100,000 in Denmark at January 1, 1961–2015. MTC, medullary thyroid carcinoma; SMTC, sporadic MTC; HMTC, hereditary MTC..

The average annual percentage change from January 1, 1998, to January 1, 2015, for all MTC, SMTC and HMTC was 2.8 (95% CI: 2.1–3.6; *P* < 0.001), 3.8 (95% CI: 2.8–4.8; *P* < 0.001), 1.5 (95% CI: 0.4–2.6; *P* = 0.010), respectively (Fig. 2).

## Discussion

In the nationwide period of this study, we report of a statistically non-significant increase in incidence for all MTC and SMTC, and similarly a non-significant decrease for HMTC. Prevalence increased significantly from 1998 to 2015.

### Limitations

To estimate the true number of SMTC and HMTC patients during a given time period, every MTC patient is preferably molecularly classified. This was the case for 182 of 224 (81%) patients in our nationwide period from 1997 to 2014 (Table 2). Of the remaining 42 (19%) patients, four were diagnosed at autopsy, while 21 were dead and 17 were alive at October 1, 2017. Ideally, *RET* testing would be offered to those alive and performed in normal tissue from the deceased (33). However, this is a burdensome affair associated with substantial ethical challenges. Instead, classification was based on relatedness to *RET* positive families by pedigree comparison along with MEN2 features other than MTC and the presence of MEN2 family history in the vast majority of molecularly unclassified patients (Table 2).

When comparing the two inclusion periods, 1960–1996 and 1997–2014, the former is >twice as long as the latter, but include an almost identical number of patients. This could reflect an increasing incidence or poor registration in the early years. To comply with the latter and ensure complete coverage, we primarily focused on the years 1997–2014, even though registration to the two nationwide registries, the Danish Cancer Registry and the Danish Thyroid Cancer Database, has been mandatory since 1987 and 1996, respectively (9, 10).

### Demographics

In the nationwide period, the female-to-male ratio for all MTC was 1.43. This is in accordance with the ratio found in other epidemiologic studies on the subject (8, 35, 36, 37, 38). As for virtually all sporadic thyroid cancers, this sex disparity still remains unexplained (39). The mean age at diagnosis of 52.4 years for all MTC patients was also comparable to that of other population-based series (8, 37). If only considering patients diagnosed at autopsy, female-to-male ratio and age at diagnosis were identical to other series (40).

### Incidence

The mean age-standardized incidence of all MTC from 1997 to 2014 did not differ significantly from that reported by large-scale studies with roughly equivalent inclusion periods (4, 7). One could have expected a higher incidence of MTC in Denmark, due to the *RET* C611Y founder effect (41). This may in part be explained by the absence of MTC in 49% (29/59) of the Danish C611Y carriers thyroidectomized during the same period. Additionally, the distribution of *RET* germline mutations in Denmark and their corresponding MTC risk level need to be taken into account. Thus, compared to other populations, Denmark has a sparse representation of codon 634 mutation carriers, whose mutations are categorized in the ‘high’ risk level, while having a high representation of C611Y carriers, whose mutation is categorized in the ‘moderate’ risk level (1, 13). Accordingly, the MTC of a C611Y carrier is more likely to pass unrecognized compared to that of a codon 634 mutation carrier, potentially influencing the MTC incidence. Also, it is possible that MTC diagnosis is more easily avoided by prophylactic thyroidectomy in C611Y carriers as MTC is believed to develop later than in codon 634 mutation carriers.

In the present study, we found no significant change in incidence of all MTC between 1997 and 2014. Similarly, studies including populations from Brazil, the USA, the Netherlands, Italy and France also failed to detect a significant change during roughly comparable periods (38, 42, 43, 44, 45). However, two recent USA studies based on Surveillance, Epidemiology, and End Results 13 data, reported a significant increase in all MTC from 1992/1993 to 2012 (5, 7). One of the studies reported a 1.87% average annual change in incidence from 1993 to 2012, but did not elaborate on the number of MTC patients (5). The other study included 1579 MTC patients and computed a 2.3% average annual incidence change from 1992 to 2012 (7). After age standardization to the 2000 USA population for suitable comparison, the average annual percentage change for all MTC in Denmark in the corresponding period was 0.7 (95% CI: −1.7 to 3.2; *P* = 0.559). The absence of significant change in Denmark may well be a question of sample size.

For the first time since *RET* testing has become available, we have calculated the mean annual incidence for SMTC and HMTC. This has been done only once before *RET* testing became available. Thus, a Swedish nationwide study, covering the period from 1970 to 1981, reported the mean age-standardized incidence of SMTC and HMTC as 0.15 and 0.06 per 100,000 per year, respectively (40). During the same period, the mean age-standardized (1970 Sweden) incidences in Denmark for SMTC and HMTC were 0.12 (95% CI: 0.09–0.15) and 0.03 (95% CI: 0.02–0.05) per 100,000 per year, respectively. One might have expected a higher incidence in Denmark compared to Sweden due to *RET* C611Y founder effect in Denmark (41). However, potential differences in the Danish and Swedish coverage in this period hinder reasonable conclusions.

The present study computed time trends for the incidence of SMTC and HMTC for the first time and showed an average annual change of 2.8% for SMTC and −3.1% for HMTC in the nationwide period. Systematically performed prophylactic thyroidectomy in two large C611Y families during the late 1970s and 1980s, one of which has been described earlier (30), may have precipitated or excluded several future MTC diagnoses, thus potentially contributing to the decreasing trend for HMTC incidence. A decreasing trend for MEN2 incidence could have a similar effect. This, however, seems less conceivable as the incidence of *RET* mutation carriers over time has been reported as either stable or increasing (46). Focusing only on prophylactic thyroidectomies performed from 1997 to 2014, little points toward a decreasing effect on temporal trends in HMTC incidence. In fact, based on the Danish *RET* cohort, the frequency of MTC in prophylactic thyroidectomized MEN2 patients did not change significantly from 47% (22/47) during 1997–2005 to 63% (20/32) during 2006–2014. However, to accurately assess the effect of prophylactic thyroidectomy on HMTC incidence, our period of complete nationwide data is too short.

Although non-significant, the opposing temporal trends in SMTC and HMTC incidence could indicate that the temporal change of 1.0% in all MTC is driven by the SMTC group rather than the HMTC group. While remaining speculative, this may also apply for the large-sample studies finding a significant increase in MTC incidence over time (3, 5, 6, 7, 8). This is supported by the significant increase in incidence reported for all major histological subtypes of non-hereditary thyroid cancer besides the anaplastic (5, 6).

### Prevalence

At January 1, 2015, the point prevalence for all MTC was 3.8 per 100,000. To the best of our knowledge, the prevalence of MTC was unknown before this publication (18). However, the Orphanet has reported an estimated prevalence of 1–9 per 100,000 (www.orpha.net). Our prevalence lies within this estimate, but in the lower end.

We found a significant increase in prevalence for all MTC from 1998 to 2015. As the incidence did not change significantly in this period, a likely explanation could be an improvement in survival. After dichotomizing the nationwide period into two equal halves, no difference was seen in overall survival (*P* = 0.573, log-rank test). Thus, the prevalence increase does not seem to be explained by a recent improvement in survival. However, comparison of patients diagnosed in the two periods, 1960–1996 and 1997–2014, demonstrated an improved overall survival over time (*P* < 0.001, log-rank test) that may potentially explain the increasing prevalence. Admittedly, one has to keep in mind the limitations of this comparison due to the potential disparity in period coverage. However, an improved survival in MTC patients during the last four decades has been seen in other studies as well (8).

As 57% of MTC patients are not biochemically cured upon initial surgery and additional 5% develop biochemical recurrence later (47), the majority of MTC patients will require life-long follow-up due to hypercalcitonemia. The increasing prevalence, therefore, implicitly suggests that the number of MTC patients needing life-long follow-up is growing significantly, warranting increased attention to the management of this patient group.

## Conclusion

We found no significant change in the incidence of all MTC, SMTC and HMTC possibly due to our small sample size. However, due to an increasing trend in the incidence of all MTC and opposing trends of SMTC (increasing) and HMTC (decreasing) incidence, it seems plausible that an increase for all MTC seen by others may be driven by the SMTC group rather than the HMTC group.

## Supplementary Material

Supplementary Material

Supplementary Figure 1

## Declaration of interest

The authors declare that there is no conflict of interest that could be perceived as prejudicing the impartiality of the research reported.

## Funding

This work was supported by the University of Southern Denmark, the Region of Southern Denmark, Odense University Hospital, Copenhagen University Hospital, the Danish Cancer Society, the Danish Cancer Research Foundation and the A.P. Moeller Foundation. The research salary of Ulla Feldt-Rasmussen is sponsored by an unrestricted research grant from the Novo Nordic Foundation.

## Author contribution statement

J S Mathiesen conceived and coordinated the study, collected data, performed statistical analyses and drafted the manuscript. S Möller performed statistical analyses and drafted the manuscript. J P Kroustrup, P Vestergaard, K Stochholm, P L Løgstrup, Å K Rasmussen, U Feldt-Rasmuseen, S Schytte, S C Londero, H B Pedersen, C H Hahn, B D Djurhuus, J Bentzen, M Gaustadnes, M Rossing, F C Nielsen, K Brixen, A L Frederiksen and C Godballe participated in data collection, and drafting of the manuscript.
