# Supplementary material for: Incidence and prevalence of sporadic and hereditary MTC in Denmark 1960–2014: a nationwide study
Source: Endocr Connect. 2018 May 14;7(6):829–39. doi: 10.1530/EC-18-0157 (PMC6000757; doi:10.1530/EC-18-0157)
Supplement: Supplementary Material [file ec-7-829-s001.pdf]

Identification of relevant cohorts

Cohorts from the Danish Pathology Register included patients histologically or cytologically diagnosed between September 1968 and December 2014 and were created using the following SNOMED codes: PHEO cohort (T93000; T93010; T93020; T93100; T93110; T93120; T93130; T93200; T93300 and M87000; M87001; M87003; M87004; M87006; M87007; M87009), HPTH cohort (T97000; T97100; T97200; T97300; T97400; T97800 and M72000; M72001; M72003; M72005; M72009; M72010; M72030; M72031; M72032; M83210; M81400), Hirschsprung cohort (S62590), MEN2A cohort (S29030) and MEN2B cohort (S29040). In the HPTH cohort 317 patients did not fulfil the exact topographical and morphological code combination specified and were therefore excluded. Thus, the PHEO cohort, HPTH cohort, Hirschsprung cohort, MEN2A cohort and MEN2B cohort comprised 482, 7,361 , 358, 10 and 3 unique patients, respectively.

Cohorts from the Danish National Patient Registry included patients coded between 1976 and 2014 and were created by using the following International Classification of Diseases (ICD)-8 and -10 codes: PHEO cohort (25529; DD350A), HPTH cohort (25200; 25201; 25209; DE210; DE210A), Hirschsprung cohort (75139; DQ431), MEN cohort (25819; DD358; DD448; DD448A) and lichen amyloidosis cohort (DL990A). The PHEO cohort, HPTH cohort, Hirschsprung cohort, MEN cohort and lichen amyloidosis cohort comprised 1,055, 14,810, 1,485, 423 and 6 unique patients, respectively.
