# Supplementary figures and images for: Incidence and prevalence of sporadic and hereditary MTC in Denmark 1960–2014: a nationwide study
Source: Endocr Connect. 2018 May 14;7(6):829–39. doi: 10.1530/EC-18-0157 (PMC6000757; doi:10.1530/EC-18-0157)

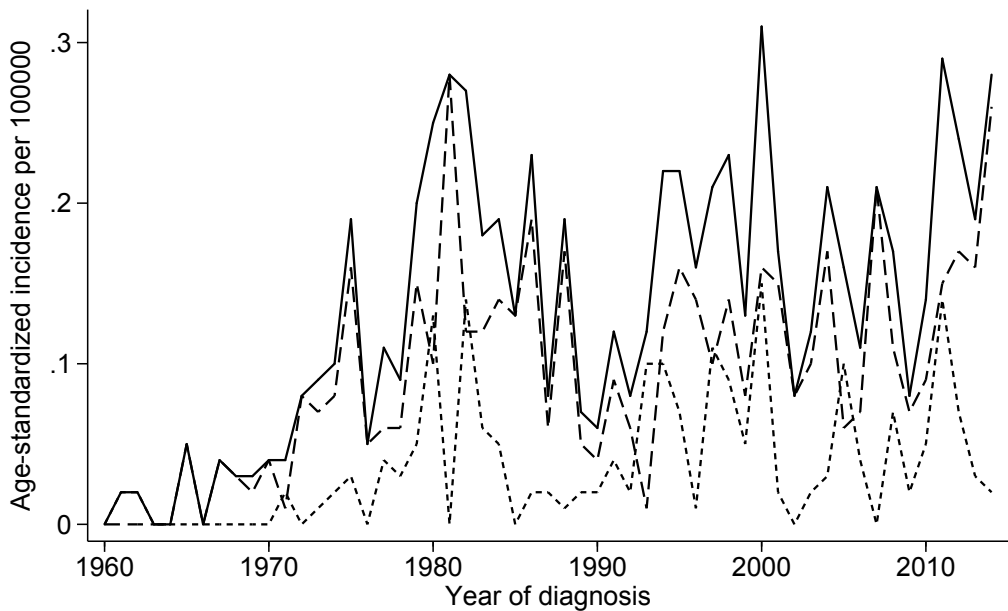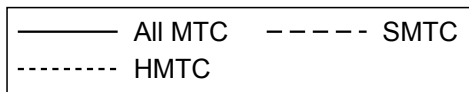

Supplement: Supplementary Figure 1 [file ec-7-829-s002.pdf]
